# Supplementary material for: Prediction Models of Primary Membranous Nephropathy: A Systematic Review and Meta-Analysis
Source: J Clin Med. 2023 Jan 10;12(2):559. doi: 10.3390/jcm12020559 (PMC9867146; doi:10.3390/jcm12020559)
Supplement: Supplementary file 1 [file jcm-12-00559-s001.zip › jcm-2107907-supplementary.pdf]

Supplementary Materials

Table S1. Literature search strategies.

Database	Strategies
Medline	((Glomerulonephritis, Membranous[MeSH Terms]) OR 'membranous nephropathy' or 'membranous glomerulonephritis'') AND ('prediction model' or 'predict* model' or 'risk score' or 'risk model' or 'model')
Embase	1. 'membranous nephropathy' or 'membranous glomerulonephritis' or 'membranous nephri*'<br>2. 'prediction model' or 'predict* model' or 'risk score' or 'risk model' or 'model'<br>3. #1 AND #2

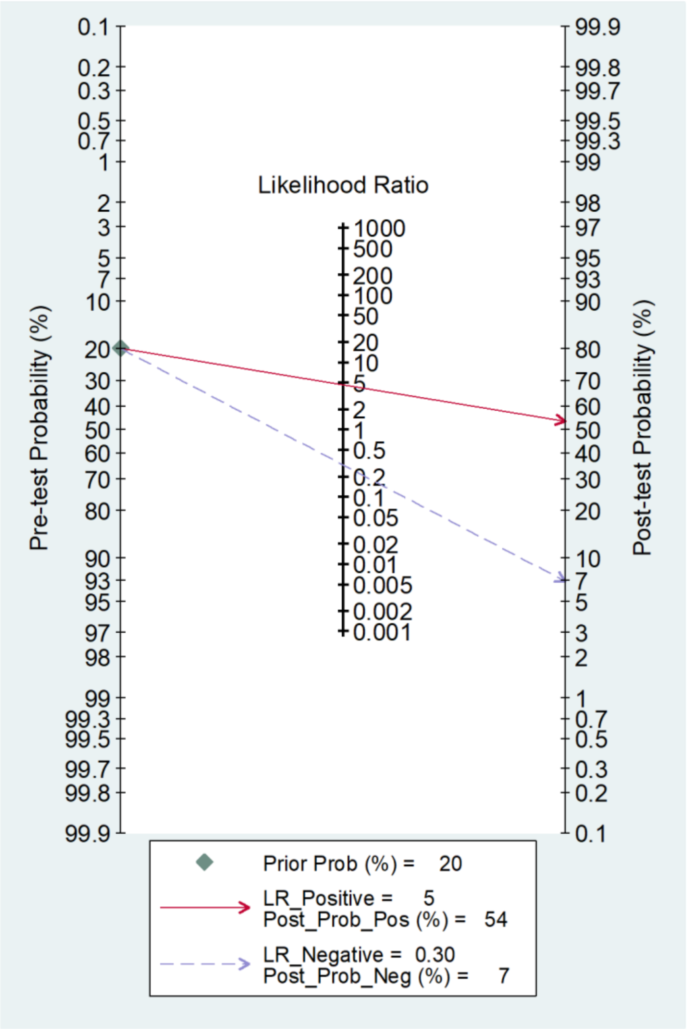

Figure S1. Fagan nomogram of the six prediction models with renal function progression as predicted outcomes.
